# Early Detection of Chemotherapy‐Induced Glucose Metabolic Alterations in Bladder Carcinoma Using Deuterium (^2^H) Metabolic Imaging: in Vitro and in Vivo Assessments

**DOI:** 10.1002/advs.202514614

**Published:** 2025-11-14

**Authors:** Lingmin Kong, Ganghan Yang, Bei Weng, Zhihua Wen, Joshua D. Kaggie, Qian Cai, Qian Wan, Cuien Zheng, Feng Du, Ye Li, Xin Liu, Ferdia A. Gallagher, Yan Guo, Shi‐Ting Feng, Chao Zou, Huanjun Wang

**Affiliations:** ^1^ Department of Radiology The First Affiliated Hospital Sun Yat‐sen University 58 Zhongshan Road 2 Guangzhou Guangdong 510080 P. R. China; ^2^ The School of Electronics and Information Engineering Harbin Institute of Technology Harbin 150001 P. R. China; ^3^ Paul C. Lauterbur Research Center for Biomedical Imaging Shenzhen Institute of Advanced Technology Chinese Academy of Sciences Shenzhen Guangdong 518055 P. R. China; ^4^ Department of Radiology University of Cambridge Cambridge CB2 0QQ UK; ^5^ Key Laboratory for Magnetic Resonance Multimodality Imaging of Guangdong Province Shenzhen Guangdong 518055 P. R. China; ^6^ State Key Laboratory of Biomedical Imaging Science, System Shenzhen Guangdong 518055 P. R. China

**Keywords:** bladder tumor, chemotherapy, deuterium, metabolism

## Abstract

Deuterium metabolic imaging (DMI) is a promising noninvasive technique for assessing tumor metabolism, yet its application in bladder cancer (BCa) remains unexplored. This study presents the first demonstration of DMI for evaluating therapeutic response in BCa through in vitro and in vivo experiments. Two types of deuterium‐labeled glucose tracers, [6,6′‐^2^H_2_]‐D‐glucose and [2,3,4,6,6′‐^2^H_5_]‐D‐glucose, are systematically compared for their efficacy in capturing glycolytic activity and treatment‐induced metabolic changes in BCa models. In subcutaneous tumors, deuterium magnetic resonance spectroscopy and spectroscopic imaging reveal pronounced suppression of glycolytic flux following chemotherapy, underscoring the potential of DMI for early therapeutic response assessment in BCa treatment. Both tracers consistently detect these chemotherapy‐related metabolism shifts in both vitro and in vivo experiments, indicating the translational advantage of [2,3,4,6,6′‐^2^H_5_]‐D‐glucose as a cost‐effective alternative to [6,6′‐^2^H_2_]‐D‐glucose. Notably, subcutaneous administration yields metabolic profiles comparable to intravenous delivery. Finally, the optimized lower‐dose, subcutaneous [2,3,4,6,6′‐^2^H_5_]‐D‐glucose protocol is successfully validated in orthotopic BCa models. These results demonstrate DMI as a reliable tool for non‐invasive monitoring of therapeutic response in BCa, with practical potential for future clinical translation.

## Graphical abstract

This study presents the first application of deuterium metabolic imaging (DMI) for assessing therapeutic response in bladder cancer. Employing [2,3,4,6,6′‐^2^H_5_]‐D‐glucose, DMI successfully identified chemotherapy‐induced suppression of glycolysis both in vitro and in vivo. The use of cost‐effective [2,3,4,6,6′‐^2^H_5_]‐glucose administered subcutaneously demonstrates promising potential for clinical translation toward noninvasive treatment monitoring.

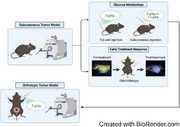

## 1 Introduction

Neoadjuvant therapies (NAT), including chemotherapy, immunotherapy, and targeted therapy, play a critical role in the management of advanced bladder cancer (BCa), offering significant survival benefits.^[^ ^1^, ^2^ ^]^ However, treatment responses are notably heterogeneous, with only a subset of patients experiencing durable benefits.^[^ ^3^, ^4^, ^5^ ^]^ Early detection of treatment responses and effective monitoring remain significant clinical challenges.

Currently, treatment responses are mainly evaluated by changes in tumor size, based on RECIST or iRECIST criteria, using conventional imaging modalities, such as CT and MRI.^[^ ^6^, ^7^, ^8^ ^]^ However, tumor size and anatomical alterations typically reflect delayed manifestations of underlying biological processes, such as metabolic dysfunction. Moreover, treatment‐induced inflammation or pseudoprogression may obscure true tumor responses, reducing the sensitivity of these modalities for early response detection.^[^ ^9^ ^]^ Most cancer cells exhibit elevated glucose uptake and lactate production, a phenomenon known as the Warburg effect.^[^ ^10^, ^11^ ^]^ By assessing differential glucose consumption, a direct and timely readout of early treatment efficacy can be obtained.^[^ ^12^, ^13^, ^14^ ^]^ This has led to an increasing emphasis on metabolic imaging as a tool for real‐time tumor monitoring.

Compared with established modalities such as positron emission tomography (PET)^[^ ^15^, ^16^ ^]^ and hyperpolarized ^13^C Magnetic Resonance Spectroscopy Imaging (MRSI),^[^ ^17^, ^18^ ^]^ deuterium metabolic imaging (DMI) stands out as a robust, simple and versatile MRSI‐based technique. DMI offers unique advantages, including the use of non‐radioactive tracers, flexible dosing and scanning protocols, along with deuterium (^2^H)‐labeled substrates like glucose and fumarate, enabling comprehensive tracing of key metabolic fluxes.^[^ ^19^, ^20^, ^21^ ^]^ In recent years, DMI has been successfully applied to characterize energy metabolism across several cancer types, such as glioblastoma,^[^ ^22^ ^]^ melanoma,^[^ ^23^ ^]^ lymphoma,^[^ ^24^ ^]^ pancreatic cancer,^[^ ^25^, ^26^ ^]^ and liver cancer,^[^ ^27^ ^]^ by quantifying glycolytic flux, evaluating telomerase reactivation, and monitoring tumor cell death. However, its application in BCa remains unexplored. Despite these promising applications, the clinical translation of DMI remains constrained by the high cost of ^2^H‐labeled substrates, with a typical dose of [6,6′‐^2^H_2_]‐glucose costing up to 1000 USD per person.^[^ ^28^ ^]^ The potential of more affordable ^2^H‐labeled glucose tracers with higher labeling efficiency has not yet been thoroughly evaluated across a broad range of cancer types. Additionally, in preclinical studies, technical challenges such as tail vein catheterization in mice, which requires extensive operator training, have low procedural success rates and can lead to cumulative vein damage, hindering their utility in longitudinal imaging studies. Subcutaneous injection offers a simpler and potentially more reproducible alternative,^[^ ^29^ ^]^ however, its feasibility for DMI has not been thoroughly evaluated. These gaps highlight the need to optimize tracer selection and delivery methods for broader DMI applications, particularly in BCa.

The current study explores the potential of DMI to assess tumor metabolism and early chemotherapeutic response in BCa. We previously introduced [2,3,4,6,6′‐^2^H_5_]‐D‐glucose as a cost‐effective tracer with performance comparable to [6,6′‐^2^H_2_]‐D‐glucose in glioma models.^[^ ^30^ ^]^ Building upon this work, we further evaluated subcutaneous (s.c.) injection of this tracer as a practical alternative to tail vein injection for longitudinal DMI in small‐animal models. In vitro, [2,3,4,6,6′‐^2^H_5_]‐D‐glucose effectively characterized metabolic activity in BCa and detected chemotherapeutic responses within 24 h post‐treatment. Additionally, s.c. Administration provides a straightforward, consistent, and minimally invasive method for tracer delivery, facilitating reliable substrate uptake. Leveraging this refined protocol, both ^2^H MRS and MRSI effectively visualized glucose metabolism in vivo, offering a reliable, noninvasive approach for early therapeutic monitoring with potential utility in guiding personalized treatment strategies.

## 2 Results

### 2.1 Metabolic Characterization of BCa Cells in Vitro

**Figure 1:**
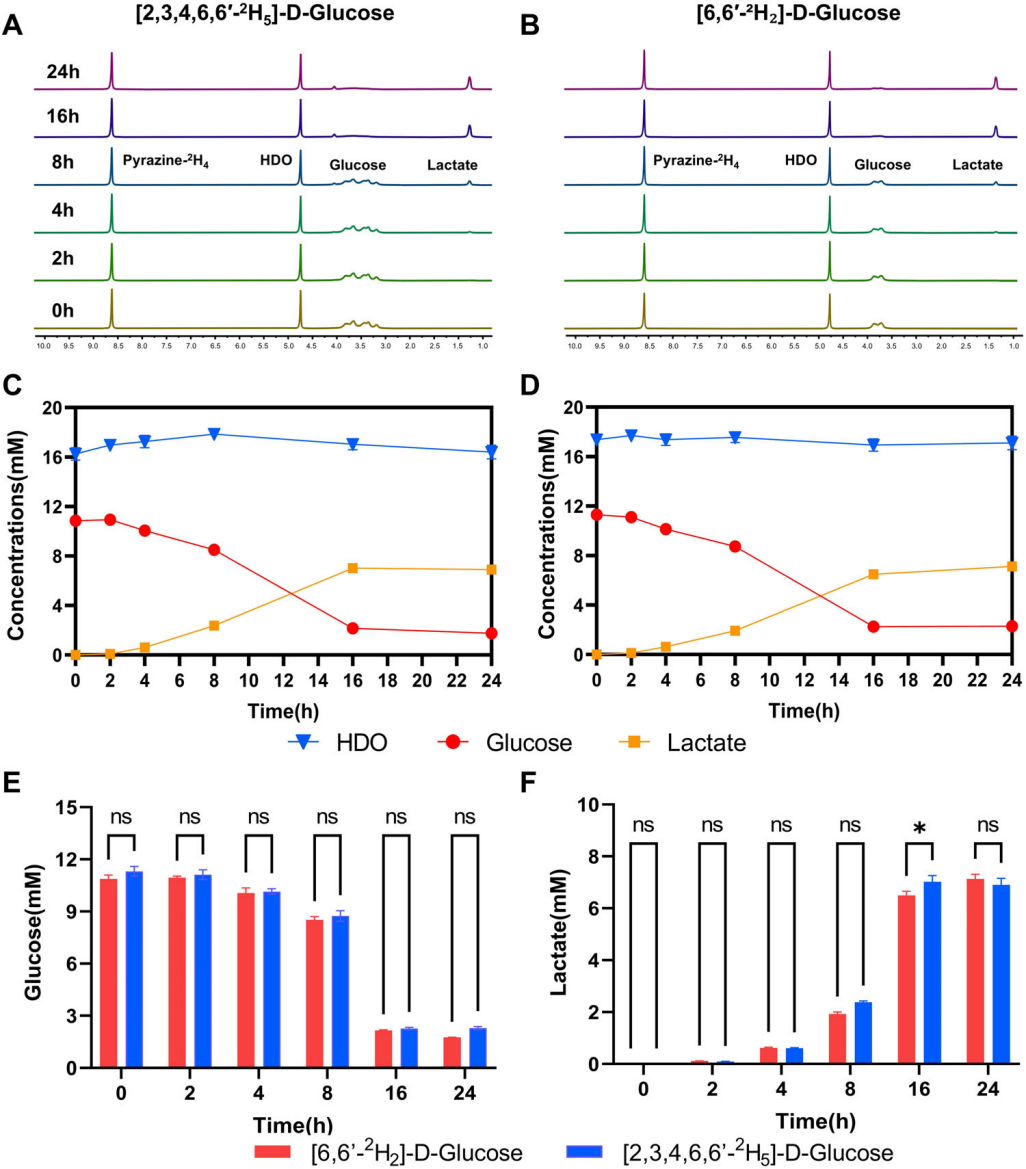
Characterization of glucose metabolism activity in bladder cancer (MB49) cells in vitro. A,B) Representative ^2^H NMR spectra of MB49 cell culture medium after incubation with [2,3,4,6,6′‐^2^H_5_]‐D‐glucose (n = 18, 3 per time point) (A) and [6,6′‐^2^H_2_]‐D‐glucose (n = 18, 3 per time point) (B); C,D) Time‐course curves showing the concentrations of deuterium‐labeled water, glucose, and lactate at multiple time points following incubation with [2,3,4,6,6′‐^2^H_5_]‐D‐glucose (C) and [6,6′‐^2^H_2_]‐D‐glucose (D); E,F) Comparison of glucose (E) and lactate (F) concentrations between the two tracers at corresponding time points. The metabolic profiles of the two tracers were largely similar. ns, not significant; ^*^, *p* < 0.05.

**Figure** **1** illustrates the concentration of ^2^H‐labelled metabolites following incubation with [2,3,4,6,6′‐^2^H_5_]‐D‐glucose and [6,6′‐^2^H_2_]‐D‐glucose at various time points (0, 2, 4, 6, 8, 16, and 24 h). Signals were detected from deuterated water, the injected glucose, and downstream lactate (Figure 1A,B). Both tracers showed a decline in deuterium‐labeled glucose concentration at the 2‐h time point, with an accumulation of deuterium‐labeled lactate that reached steady‐state levels by 16 h (Figure 1C,D). Across most time points, no significant differences were observed between these two deuterated agents (Figure 1E,F). Although not statistically significant, [2,3,4,6,6′‐^2^H_5_]‐D‐glucose and its downstream metabolite ^2^H‐lactate tended to produce higher signal intensities compared to [6,6′‐^2^H_2_]‐D‐glucose and its corresponding metabolites (Figure 1E,F).

### 2.2 Deuterated Glucose Metabolism Detects Cell Death in BCa Cells in Vitro

**Figure 2:**
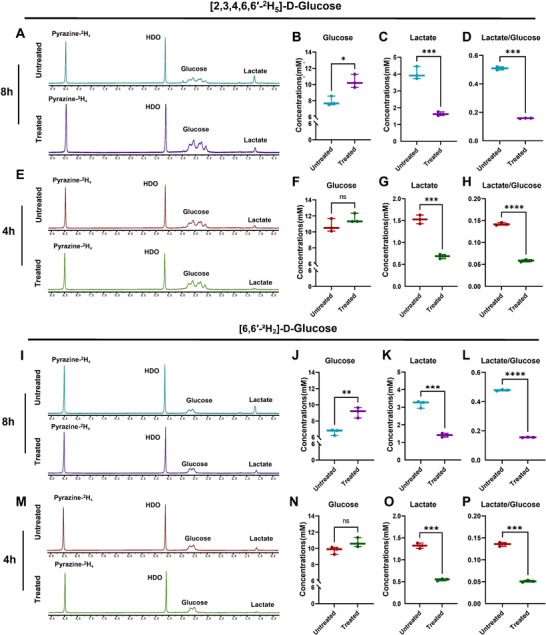
In vitro characterization of chemotherapy‐induced effects on glucose metabolism. A,E,I, and M) Representative ^2^H NMR spectra of media samples collected at 4 and 8 h from control (n = 3, per group) and treatment (n = 3, per group) groups, respectively; B–D, F–H, J–L and N–P) Chemotherapy significantly suppresses glycolytic metabolism in MB49 cells, as evidenced by reduced downstream ^2^H‐lactate production. ns, not significant; ^*^, *p* < 0.05; ^**^, *p* < 0.01; and ^***^, *p* < 0.001.

Based on our findings, MB49 cells were treated with 900 nm gemcitabine and 2.5 µg mL^−1^ cisplatin for 24 h. Compared to the control group, cell viability in the treated group decreased by 50% (Figure S1, Supporting Information). Following the incubation with [6,6′‐^2^H_2_]‐D‐Glucose or [2,3,4,6,6′‐^2^H_5_]‐D‐Glucose for 4 or 8 h, the concentrations of deuterium‐labelled glucose and lactate in the culture medium were measured using ^2^H NMR. Representative spectra are shown in **Figure** **2** A–E, I and M. When [2,3,4,6,6′‐^2^H_5_]‐D‐glucose was used as a tracer, drug‐treated cells showed significantly lower lactate concentration (*P* < 0.001, Figure 2C) and lactate/glucose ratio (*P* < 0.001, Figure 2D) than controls after 8 h of incubation (n = 3 for each time point). In addition, the residual glucose concentration in the treated cell culture was higher than that of the untreated group (*P* = 0.014, Figure 2B), indicating suppressed glucose consumption following chemotherapy. For cells incubated for 4 h, ^2^H MRS measurements of medium samples also showed a significant reduction in labeled lactate (*P* < 0.001, Figure 2G) and the lactate/glucose ratio (*P* < 0.001, Figure 2H) in treated cells, comparable to the changes observed at 8 h. However, no significant difference in glucose consumption was observed between treated and untreated groups with 4‐h incubation (*P* = 0.196, Figure 2F). Similar metabolic changes were observed in cells incubated with [6,6′‐^2^H_2_]‐D‐glucose (Figure 2J–L and N–P). Detailed quantitative data are presented in **Table** **1**.

**Table 1:** Quantification of ^2^H‐labeled glucose and downstream metabolites (lactate) in MB49 cells following incubation for four or eight hours with two different deuterated glucose tracers before and after chemotherapy treatment.

	Glucose concentrations [mm]	Lactate concentrations [mm]	Lactate/glucose
[2,3,4,6,6′‐^2^H_5_]‐glucose			
4h			
Treated	10.75 ± 0.81	0.68 ± 0.05	0.06 ± 0.01
Untreated	11.65 ± 0.60	1.53 ± 0.10	0.14 ± 0.01
*P*	.196	<.001	<.0001
8h			
Treated	10.37 ± 0.82	1.64 ± 0.11	0.16 ± 0.01
Untreated	7.92 ± 0.57	4.04 ± 0.38	0.51 ± 0.01
*P*	.014	<.001	<.001
[6,6′‐^2^H_2_]‐glucose			
4h			
Treated	10.71 ± 0.57	0.54 ± 0.02	0.05 ± 0.01
Untreated	9.76 ± 0.45	1.32 ± 0.06	0.13 ± 0.01
*P*	.088	<.001	<.001
8h			
Treated	9.07 ± 0.64	1.41 ± 0.11	0.15 ± 0.01
Untreated	6.64 ± 0.37	3.18 ± 0.24	0.48 ± 0.01
*P*	.005	<.001	<.0001

### 2.3 Glucose Metabolism in Subcutaneous Bladder Tumor Models

**Figure 3:**
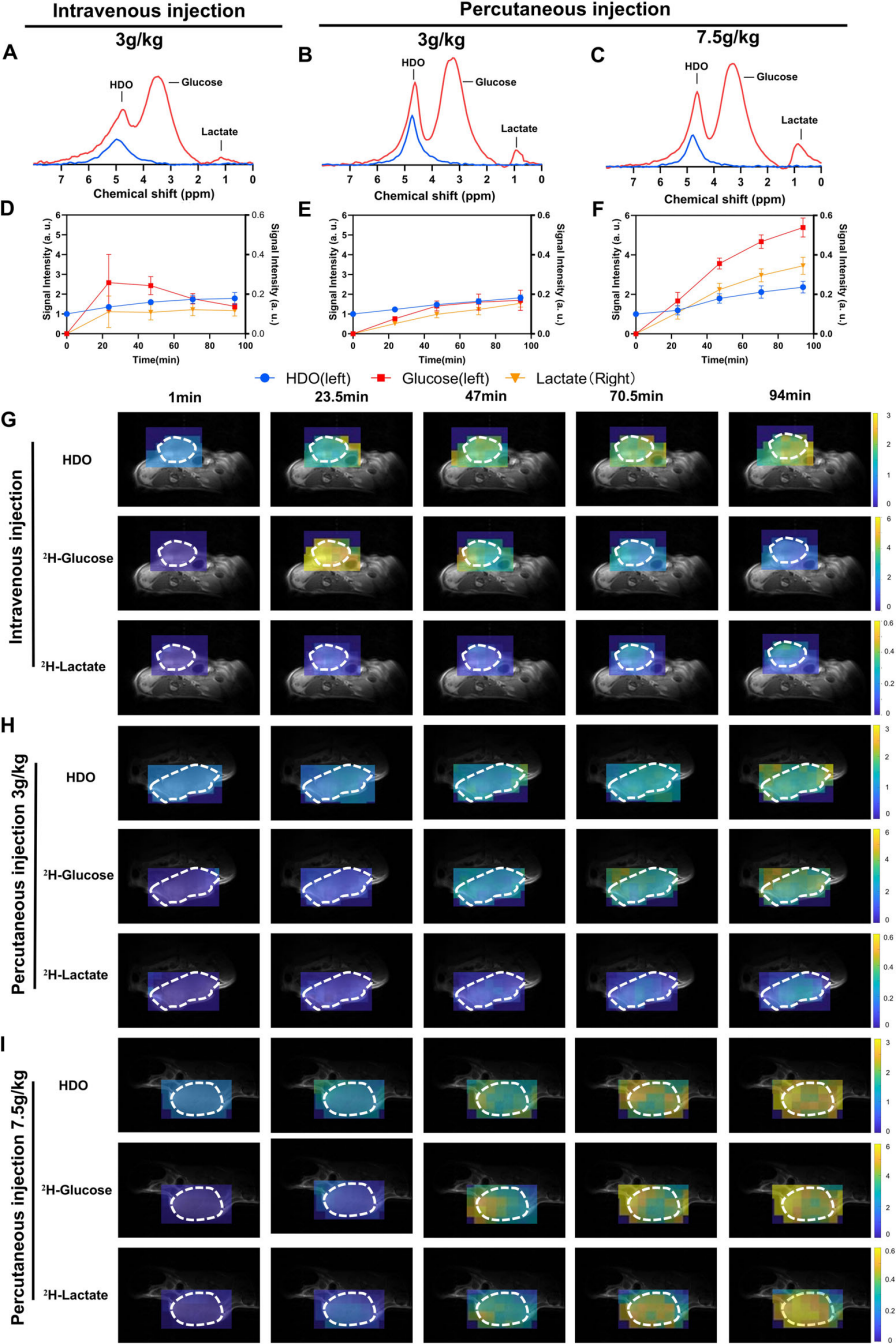
Comparison of administration routes and dosing regimens for ^2^H‐glucose delivery in subcutaneous bladder cancer models. A–C) Representative ^2^H NMR global spectra acquired from MB49 tumor–bearing mice. Mice received either an intravenous injection of [2,3,4,6,6′‐^2^H_5_]‐D‐glucose at 3 g kg^−1^ (n = 4) (A), or subcutaneous injection at 3 g kg^−1^ (n = 6) (B) and 7.5 g kg^−1^ (n = 6) (C), respectively. Blue indicates the baseline spectra, and red represents spectra acquired 94 min after the administration of deuterium‐labeled glucose; D–F) Time‐course curves of normalized ^2^H‐labeled water, glucose (**left Y‐axis**) and lactate (**right Y‐axis**) signal intensities measured at multiple time points following administration according to the three protocols corresponding to A–C; G–I) Representative ^2^H MRS imaging acquired at multiple time points following intravenous injection at 3 g kg^−1^ (G), and subcutaneous injection at 3 g kg^−1^ (H) and 7.5 g kg^−1^ (I). Tumor locations are indicated by a white dashed line on the overlaid ^1^H anatomical reference images.

When subcutaneous tumors reached 10 mm, coil‐localized ^2^H MRS spectra were acquired. Mice received either an intravenous (i.v.) injection of [6,6′‐^2^H_2_]‐D‐glucose (n = 3) or [2,3,4,6,6′‐^2^H_5_]‐D‐glucose at 3 g kg^−1^ (n  = 4), or a subcutaneous (s.c.) injections of [2,3,4,6,6′‐^2^H_5_]‐D‐glucose at 3 g kg^−1^ (n  =  6) and 7.5 g kg^−1^ (n  =  6). Compared with [6,6′‐^2^H_2_]‐D‐glucose, administration of [2,3,4,6,6′‐^2^H_5_]‐D‐glucose resulted in higher signals for glucose and deuterated water (HDO), whereas lactate signals did not differ appreciably between the two tracers (Figure S2, Supporting Information). Representative baseline and summed spectra acquired during a 94‐min period for the [2,3,4,6,6′‐^2^H_5_]‐D‐glucose experiments are shown in **Figure** **3**A–C. Temporal profiles of HDO, glucose, and lactate concentrations are presented in Figure 3D–F. Following i.v. Administration, tumor glucose concentrations peaked rapidly and then declined. In contrast, s.c. Injection caused a slower, more gradual increase in tumor glucose levels. At 3 g kg^−1^, glucose peaked at 47 min post‐injection and subsequently decreased, whereas at 7.5 g kg^−1^, glucose continued to rise throughout the observation period without plateauing. Meanwhile, HDO concentrations increased gradually across all injection routes and dosing groups (Figure S3A, Supporting Information), with no significant differences observed among them. Regarding intratumoral lactate dynamics, i.v. Injection produced significantly higher lactate concentrations during the early phase compared to both s.c. Injection at 3 and 7.5 g kg^−1^. However, by 47 min post‐injection, s.c. Administration at 7.5 g kg^−1^ resulted in markedly higher lactate levels than the other two groups (Figure S3C, Supporting Information). In addition, the lactate/glucose ratio—an indicator of glycolytic flux—was calculated over the 94‐min period following [2,3,4,6,6′‐^2^H_5_]‐D‐glucose administration. The ratios were 0.08 ± 0.06 for i.v. injection at 3 g kg^−1^, 0.08 ± 0.01 for s.c. injection at 3 g kg^−1^, and 0.07 ± 0.02 for s.c. injection at 7.5 g kg^−1^. Although numerical differences were observed, the lack of statistical significance (*P*  =  0.720) suggests that metabolic readouts remained consistent regardless of administration route or dosing regimen.

### 2.4 Detection of Treatment Response in Vivo Following Subcutaneous Injection of Deuterated Glucose

**Figure 4:**
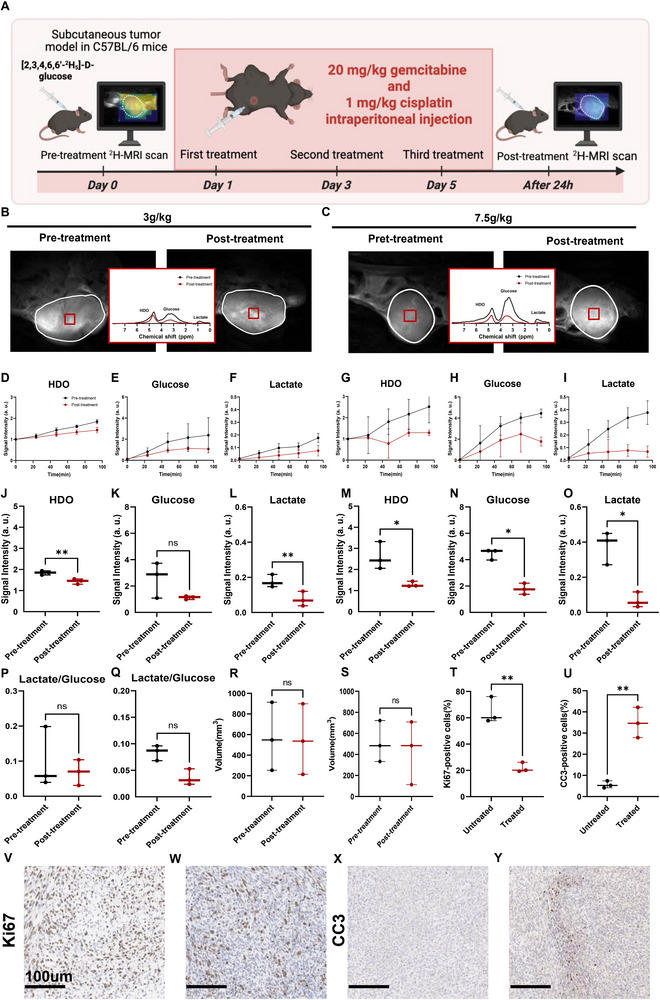
Effects of chemotherapy on glucose metabolism in subcutaneous bladder cancer models in vivo. A) Schematic overview of the experimental timeline (https://BioRender.com/9734kim); B,C) Representative ^2^H spectra from voxels within the tumor in ^2^H‐MRS images of MB49 tumor–bearing animals infused with [2,3,4,6,6′‐^2^H_5_]‐D‐glucose at doses of 7.5 g kg^−1^ (n = 3) (B) and 3 g kg^−1^ (n = 3) (C) before and after treatment. Spectra are depicted in red for pre‐treatment and black for post‐treatment. Tumor regions are outlined in white on the T2‐weighted ^1^H anatomical image, with voxel positions used for Chemical Shift Imaging (CSI) indicated by boxes. Signal intensities of labeled glucose, water, and lactate were quantified from the summed spectra and normalized to baseline water signals; D–I) Serial measurements of ^2^H‐labeled water, glucose and lactate in tumor‐bearing animals before and after treatment, following the administration of[2,3,4,6,6′‐^2^H_5_]‐D‐glucose at 3 g kg^−1^ (D–F) or 7.5 g kg^−1^ (G‐I); J–Q) Comparative analysis of ^2^H‐labeled metabolite concentrations during the 23.5–94 min post‐infusion period. Results include water (J,M), glucose (K,N), lactate (L,O) concentrations and the lactate/glucose ratio (P,Q) before and after treatment at doses of 3 g kg^−1^ (J–L,P) and 7.5 g kg^−1^ (M–O,Q), respectively; R–S) Quantitative tumor imaging measurements pre‐ and post‐treatment at doses of 7.5 g kg^−1^ (R) and 3 g kg^−1^ (S); T,U), Percentage of Ki67‐positive cells (T) and CC3‐positive cells (U) in tumor sections from the control (n = 3) and treated (n = 3) group; V–Y), Representative sections of MB49 tumors stained for Ki67 V–W) and CC3 (X,Y) in the control V–X) and treatment W–Y) groups. ns, not significant; ^*^, *p* < 0.05; ^**^, *p* < 0.01.

Baseline ^2^H‐MRSI was acquired following the administration of [2,3,4,6,6′‐^2^H_5_]‐D‐Glucose at doses of 3 g kg^−1^ (n = 3) or 7.5 g kg^−1^ (n = 3). The animals were then treated with intraperitoneal gemcitabine (20 mg kg^−1^) and cisplatin (1 mg kg^−1^) three times per week on alternate days. Post‐treatment tumor ^2^H‐MRSI was obtained with the same glucose infusion and imaging protocols. The experimental workflow is shown in **Figure** **4**A, and the ^2^H‐MRSI data obtained before and after treatment at different injection concentrations are presented in Figure 4B,C. When [2,3,4,6,6′‐^2^H_5_]‐D‐glucose was administered at a dose of 7.5 g kg^−1^, labeled lactate signal intensities were reduced by 80%, from 0.38 ± 0.09 in pretreatment animals to 0.07 ± 0.04 in the animals (*P* = 0.034, Figure 4I–O). Additionally, HDO signal intensities also decreased by 50%, from 2.60 ± 0.65 to 1.29 ± 0.13 (*P* = 0.050, Figure 4G–M). Similar changes were observed following the injection of 3 g kg^−1^ [2,3,4,6,6′‐^2^H_5_]‐D‐glucose, with a 55% reduction in labeled lactate signal intensities, from 0.18 ± 0.04 to 0.08 ± 0.04 (*P* = 0.002, Figure 4F–L), and a 22% reduction in the HDO levels, from 1.84 ± 0.10 to 1.44 ± 0.13 (*P* = 0.002, Figure 4D–J). Although a decreasing trend in tumor glucose signal intensities was observed after treatment, only the 7.5 g kg^−1^ [2,3,4,6,6′‐^2^H_5_]‐glucose group showed a statistically significant reduction in signal intensities, from 4.44 ± 0.41 to 1.78 ± 0.42 (*P* = 0.015, Figure 4H–N). No significant change was found in the 3 g kg^−1^ group (*P* = 0.204, Figure 4E,K). The lactate/glucose ratio decreased after treatment in both the 7.5 g kg^−1^ (Figure 4Q) and 3 g kg^−1^ (Figure 4P) groups; however, the changes were not statistically significant (*P* = 0.060 and 0.453, respectively). Additionally, no significant changes were observed in tumor volumes 24 h after the completion of chemotherapy (Figure 4R,S). To further evaluate changes in cell proliferation and cell death, tumor sections from the treatment and control groups were stained for Ki67 and cleaved caspase 3 (CC3). In the treatment group, tumors showed significant decreases in the percentage of Ki67‐positive cells and significant increases in CC3‐positive cells following chemotherapy (Figure 4T–Y). Changes in DMI images before and after treatment are illustrated in Figure S4 (Supporting Information).

### 2.5 Detection of Treatment Response ‐in Vivo Using Diffusion‐Weighted and Dynamic Contrast Agent‐Enhanced MRI

**Figure 5:**
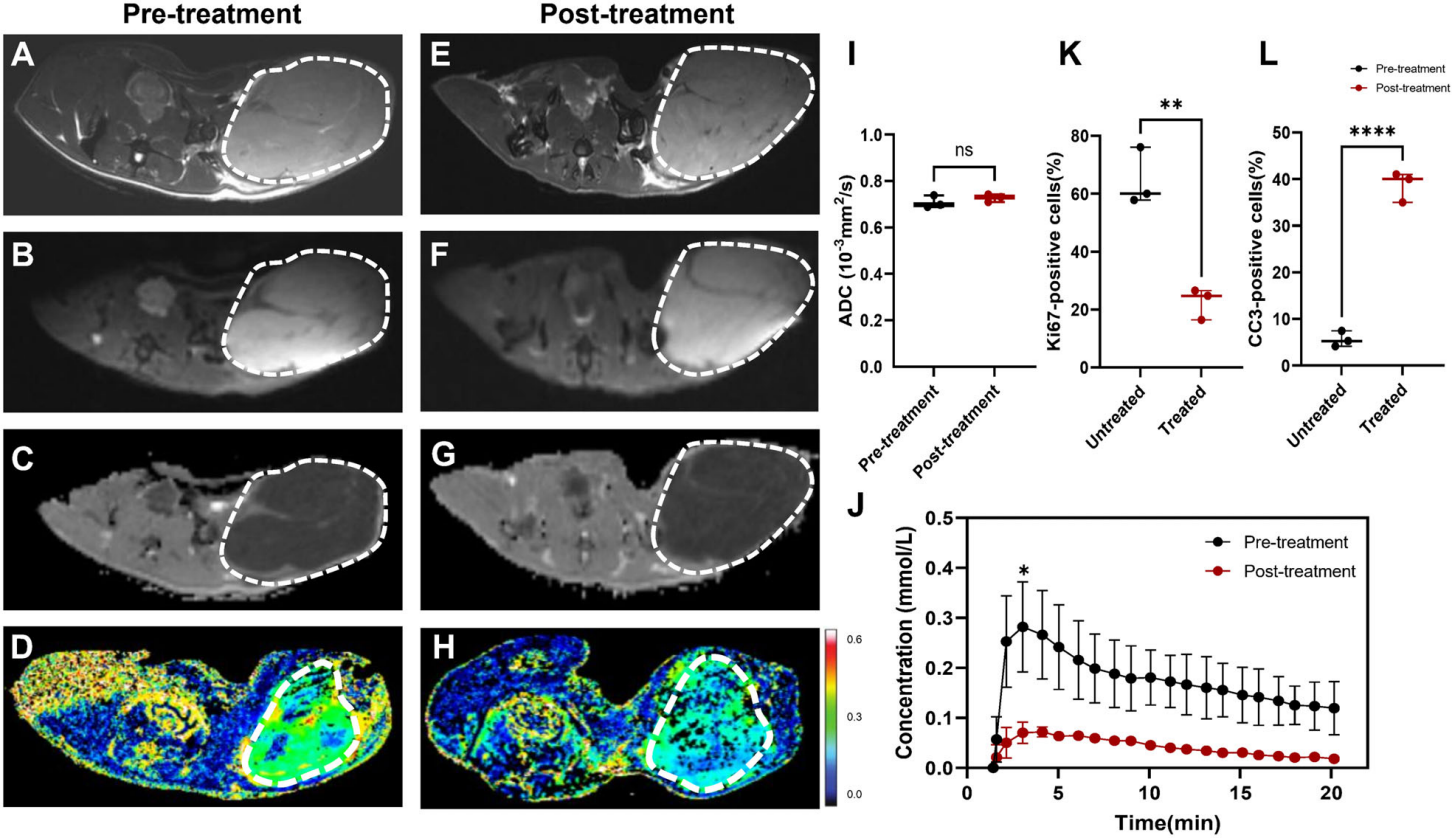
Diffusion weighted imaging and dynamic contrast agent‐enhanced MRI measurements of perfusion in subcutaneous bladder cancer models before and after treatment. Representative axial T2‐weighted images (T2WI) (A, E), diffusion weighted images (b = 1000 s/mm^2^) (B,F), apparent diffusion coefficient (ADC) maps (C, G), and color intensity maps of the mean contrast agent concentration (D, H), acquired at 9.4 T before treatment (A–D) and 24 h post‐treatment (E–H). Figure 5A–C and E–G correspond to one mouse, whereas Figure 5D–H correspond to another. Tumor locations are outlined by dotted white lines; I) Mean ADC values measured before and at 1 day after the last chemotherapy treatment in MB49 tumors (n = 3); J) Estimated contrast agent concentration in tumor tissue at the indicated time points after intravenous injection, measured before and 24 h after the last chemotherapy treatment. Data are shown as mean ± SD; Percentage of Ki67‐positive cells K) and CC3‐positive cells L) tumors in tumor sections from the control (n = 3) and treated (n = 3) group. ns, nonsignificant; *, *p* < 0.05; ^**^, *p* < 0.01;^****^, *p* < 0.0001.

Changes in tumor perfusion (n = 3) and the apparent diffusion coefficient (ADC) values (n = 3) were assessed in separate cohorts of MB49 tumor‐bearing mice (**Figure** **5**). At 24 h post‐treatment, tumors showed a slight increase in mean ADC values (0.71 × 10^−3^ mm^2^ s^−1^ ± 0.03 vs 0.73 × 10^−3^ mm^2^ s^−1^ ± 0.02, *P* = 0.116) and lower tumor contrast concentrations (0.18 mm ± 0.06 vs 0.04 mm ± 0.01, *P* = 0.059) with significantly lower concentrations observed in the first 4 min post‐contrast agent injection (*P* = 0.049) (Figure 5J).

### 2.6 Glucose Metabolism in Orthotopic Bladder Tumor Models

**Figure 6:**
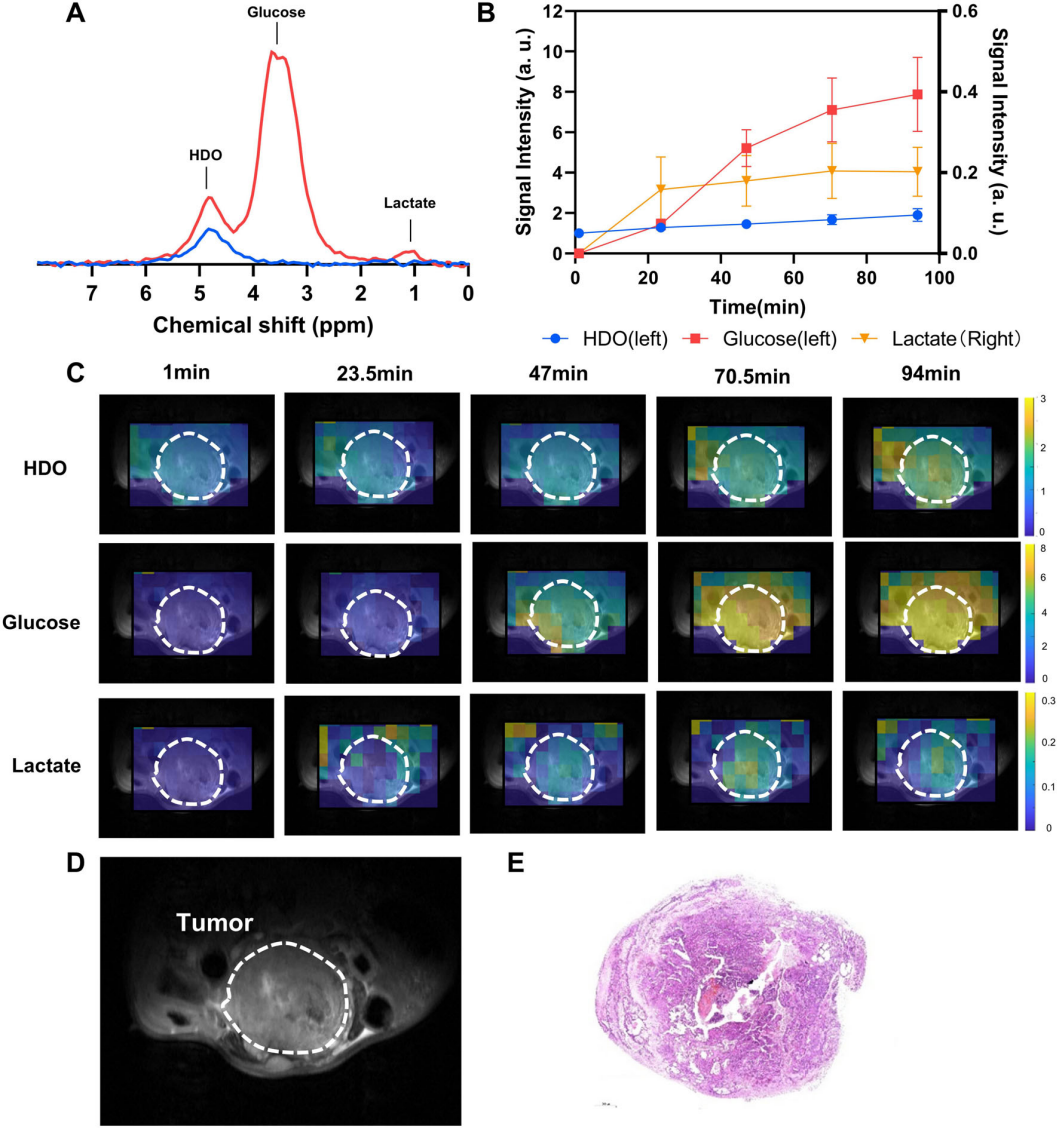
Deuterium metabolic imaging of glucose metabolism in an orthotopic bladder cancer model. A) Representative ^2^H spectrum acquired from an orthotopic MB49 tumor model (n = 4). Blue indicates the baseline spectra, and red represents spectra acquired 94 min after the administration of deuterium‐labeled glucose; B) Time‐course curves showing the levels of ^2^H‐labeled glucose, lactate, and water at multiple time points post‐injection; C) Dynamic ^2^H MRS images acquired at different time points following tracer administration, demonstrating the spatial distribution of labeled metabolites in the tumor‐bearing bladder; D) Corresponding anatomical T2‐weighted ^1^H image showing the bladder and surrounding structures, with the tumor region delineated by a white dashed line; E) Hematoxylin and eosin (H&E) staining of a bladder tissue confirming tumor localization.

Based on our findings, we implemented the optimal injection protocol in orthotopic BCa models. Mice were implanted orthotopically with MB49 cells in the posterior wall of the bladder (n = 4). 3D ^2^H chemical shift images were obtained following a s.c. injection of 3 g kg^−1^ [2,3,4,6,6′‐^2^H_5_]‐D‐glucose from animals with MB49 tumors. Representative spectra covering the entire tumor are shown in **Figure** **6**A. In orthotopic tumors, intratumoral glucose levels reached a steady state at ≈70.5 min post‐injection, while lactate and HDO levels gradually increased over time (Figure 6B). Compared to subcutaneous tumors, orthotopic tumors exhibited a delayed glucose peak but significantly higher intratumoral glucose accumulation, indicating differences in glucose transport between the two tumor models. However, lactate and HDO levels showed no significant differences between the two models (Figure S5, Supporting Information).

## 3 Discussion

This study demonstrated the feasibility of using DMI with [2,3,4,6,6′‐^2^H_5_]‐D‐glucose to noninvasively quantify tumor metabolism and assess early therapeutic responses in BCa models. We established a simplified protocol that uses s.c. Injection instead of i.v. Administration, thereby reducing complexity while effectively delivering the tracer. Both 3 and 7.5 g kg^−1^ s.c. Doses successfully detected treatment‐induced metabolic alterations, with higher sensitivity observed at the 7.5 g kg^−1^ dose. Importantly, validation in the orthotopic BCa model highlights the protocol's potential for broader application of DMI in preclinical and clinical oncology.

DMI is an emerging, minimally invasive metabolic imaging technique that has been used to visualize real‐time metabolic activity in several tumors, including brain,^[^ ^31^, ^32^ ^]^ liver,^[^ ^27^ ^]^ breast^[^ ^33^ ^]^ and pancreatic^[^ ^25^, ^26^ ^]^ cancers, using isotope‐labelled substrates. Compared to other isotope‐based methods, such as hyperpolarized ^13^C MRI, DMI offers several advantages, including technical simplicity, acquisition robustness, and lower operational costs.^[^ ^34^, ^35^ ^]^ However, the relatively high expense of deuterated substrates remains a major barrier to widespread clinical translation. In previous work, we introduced [2,3,4,6,6′‐^2^H_5_]‐D‐glucose as a cost‐effective and metabolically equivalent alternative to [6,6′‐^2^H_2_]‐D‐glucose in glioma models.^[^ ^30^ ^]^ Deuteration of the glucose molecule does not alter its metabolic pathway. Both tracers are phosphorylated by hexokinase and follow the canonical glycolytic pathway, releasing deuterium into body water at multiple enzymatic steps, including those catalyzed by triosephosphate isomerase, glyceraldehyde‐3‐phosphate dehydrogenase, and lactate dehydrogenase. Chang et al.^[^ ^23^ ^]^ proposed to the use of [^2^H_7_]‐D‐glucose in melanoma studies. Although the additional labeling at C1 would further increase total HDO yield, it would also substantially increase the synthesis cost of this deuterated substrate. In our study, the extra deuterium labeling in [2,3,4,6,6′‐^2^H_5_]‐D‐glucose does not alter its metabolic fate compared with [6,6′‐^2^H_2_]‐D‐glucose but increases the number of detectable nuclei, resulting in comparable lactate signals and improved HDO signals with a higher signal‐to‐noise ratio.^[^ ^23^ ^]^ Importantly, the synthesis of [2,3,4,6,6′‐^2^H_5_]‐D‐glucose reduces probe costs by nearly an order of magnitude compared with commercially available [6,6′‐^2^H_2_]‐D‐glucose. This dramatic cost advantage not only makes large‐scale metabolic imaging studies more feasible but also substantially enhances the translational potential of this probe for clinical applications.

Building on this foundation, the current study is the first to apply [2,3,4,6,6′‐^2^H_5_]‐D‐glucose in BCa, with systematic evaluation conducted both in vitro and in vivo. Our results showed no significant difference in glucose consumption or lactate production between the two tracers at most time points (*P* > 0.05). However, a slight variation in lactate levels was observed at 18 h (*P* = 0.042), possibly reflecting delayed‐phase metabolic variation or mild metabolic exhaustion during prolonged incubation. Importantly, chemotherapy‐induced metabolic reprogramming, characterized by decreased utilization of ^2^H‐labeled glucose and a significant reduction in ^2^H‐lactate signals, was equally detectable with both tracers, supporting their interchangeable use in BCa studies. In vivo, [2,3,4,6,6′‐^2^H_5_]‐D‐glucose provided clear metabolic contrast in subcutaneous BCa models, with chemotherapy‐treated tumors showing significant reductions in ^2^H‐glucose uptake, as well as decreases in ^2^H‐lactate and HDO production (*P* < 0.01). Notably, quantitative parameters derived from DCE and DWI MRI exhibited no significant alterations, and tumor volume remained largely unchanged during the treatment period, highlighting DMI's sensitivity over anatomical imaging for early therapy assessment. Additionally, [2,3,4,6,6′‐^2^H_5_]‐D‐glucose offers an improved spectral signal‐to‐noise ratio (SNR) due to its additional deuterium labeling. Coupled with its lower synthesis cost and greater accessibility, this tracer represents a promising and scalable alternative for DMI‐based metabolic imaging across diverse tumor models.^[^ ^36^ ^]^

In addition, we assessed various administration routes and dosages of [2,3,4,6,6′‐^2^H_5_]‐D‐glucose in vivo. Consistent with previous reports,^[^ ^29^ ^]^ our findings confirmed that s.c. Injection is a practical alternative to i.v. Delivery. While glucose uptake in tumor regions varied between administration routes, downstream metabolic indicators such as HDO production and the lactate‐to‐glucose ratio remained consistent, suggesting that both delivery strategies are equivalent. Unlike i.v. Injection, which requires skilled personnel and invasive vascular access, s.c. Administration is technically straightforward, requires minimal operator training, and is better tolerated by experimental animals, facilitating its use in longitudinal imaging studies. In previous studies,^[^ ^29^ ^]^ high‐dose s.c. Injections (1 m, 25ul g^−1^) were commonly employed to counter the slower systemic absorption associated with this route, thereby achieving a satisfactory intratumoral signal. In the current study, we further evaluated the feasibility of a lower dose (3 g kg^−1^) comparable to i.v. Injection. Notably, this reduced dose effectively maintained metabolic contrast, showing promise as a viable alternative with reduced physiological burden. Dose‐dependent effects were evident at later time points: while the 7.5 g kg^−1^ dose increased intratumoral lactate levels and sensitivity for detecting glycolytic activity, both the 7.5 and 3 g kg^−1^ doses effectively captured metabolic alterations pre‐ and post‐chemotherapy. The 3 g kg^−1^ dose may also be more practical for clinical use, balancing imaging quality with cost, injection volume, and patient tolerability. Collectively, these findings endorse s.c. Injection as a reliable, scalable, and minimally invasive method for DMI, simplifying experimental workflows, reducing technical complexity, and enhances applicability in research.

To further investigate the suitability of the optimized injection protocol in orthotopic BCa models, we used the 3 g kg^−1^ subcutaneous injection strategy. Despite the reduced tumor volume and deeper anatomical location compared to subcutaneous xenografts, a sufficient deuterium signal was obtained to measure intratumoral glucose metabolism. Notably, orthotopic tumors exhibited a delayed glucose peak and higher glucose retention compared to subcutaneous tumors, with no significant differences in lactate or HDO levels (all *P* > 0.05). These findings suggest that variations in tumor microenvironment—such as vascular density, perfusion dynamics, or tissue architecture—may influence glucose delivery in orthotopic models.^[^ ^37^, ^38^ ^]^ The consistent lactate and HDO levels between the two settings reinforce the robustness of DMI in monitoring glycolytic activity and deuterium exchange across different anatomical and physiological tumor contexts, supporting its promising use for noninvasive metabolic tracking in BCa models. In conclusion, this study demonstrates that [2,3,4,6,6′‐^2^H_5_]‐D‐glucose is a reliable and cost‐effective tracer for DMI, capable of effectively capturing key metabolic features in BCa. The adoption of s.c. Iinjection not only simplifies the administration procedure but also enable reduced glucose dosing without compromising imaging quality.

## 4 Experimental Section

### Cell Line and Cell Culture

MB49 cells were cultured in DMEM medium supplemented with L‐glutamine, 10% fetal bovine serum (FBS), 100 U mL^−1^ penicillin, and 100 mg mL^−1^ streptomycin. The culture was maintained in an incubator with a humidified atmosphere containing 5% CO_2_ at 37 °C.

### NMR Spectroscopy of Extracellular Media Samples

For media extract analysis, MB49 cells (1 × 10^6^) were seeded in non‐coated T25 flasks and cultured for 3 days. After removing the medium, the cells were washed with PBS and incubated in 5 mL of glucose‐free DMEM containing 10 mmol L^−1^ of either [6,6′‐^2^H_2_] ‐D‐Glucose (Sigma–Aldrich, USA) or [2,3,4,6,6′‐^2^H_5_]‐D‐Glucose (Dingbang Biotechnology Co., China) for 0, 2, 4, 8, 16, and 24 h. At each time point, the entire supernatant from each individual sample was collected, concentrated using a Savant SpeedVac (Thermo Scientific), and dissolved in 600 uL of PBS. For all samples, ^2^H_4_‐pyrazine (Toronto Research Chemicals, Canada) was added as an internal reference at a final concentration of 5 mm. Finally, 475 µL of the reconstituted sample was transferred into an NMR tube and mixed with 25 µL of a 100 mm ^2^H_4_‐pyrazine solution, resulting in a final volume of 500 µL. For the treatment group, MB49 cells were pretreated with 900 nm gemcitabine and 2.5 µg mL^−1^ cisplatin for 24 h, followed by PBS washing and incubation with the same deuterated glucose‐containing medium for 4 and 8 h. The medium samples were processed as described above. Cell viability was assessed using the CCK8 assay. ^2^H nuclear magnetic resonance (NMR) spectra were acquired at 310 K using the ^2^H coil of a 5‐mm ^1^H/broadband inverse detection probe in a 14.1 T NMR spectrometer (Bruker Spectrospin Ltd.) with a 90° pulse, a repetition time (TR) of 3 s, and a spectral width of 2,000 Hz, captured into 1024 datapoints, with the sum of 1024 transients. The concentrations of all metabolites were calculated from peak integrals based on the internal ^2^H_4_‐pyrazine reference.

### Animal Preparation

In this study, both subcutaneous and orthotopic bladder tumor models were established. For the subcutaneous bladder tumor model, MB49 cells (1 × 10^6^) were resuspended in ice‐cold PBS and implanted into C57BL/6J mice (6–8 weeks of age; Guangdong Medical Laboratory Animal Center) by subcutaneous injection in the lower flank. For the orthotopic bladder tumor model, six‐week‐old C57BL/6J mice (18–22 g) were anaesthetized with an intraperitoneal injection of 150 µL of 0.05% isoflurane. Mice were placed in the supine position and immobilized on the surgical board. Following bladder exposure, 10 µL of MB49 cell suspension (2 × 10^5^) was injected directly into the posterior bladder wall, and the abdominal wall was subsequently sutured. All mice were housed in a specific pathogen‐free (SPF) environment with free access to standard food and water. This study was approved by the Animal Ethics Committee of the First Affiliated Hospital of Sun Yat‐sen University, China ([2024]085).

### Chemotherapy Treatment

Six subcutaneous bladder tumor‐bearing mice were randomly assigned to experimental groups. Treatment was initiated once the tumor reached a size of 10 mm. Mice received intraperitoneal injections of gemcitabine (10 mg kg^−1^) and cisplatin (1 mg kg^−1^) three times per week.

### ^2^H MR Spectroscopy and Spectroscopic Imaging In Vivo

Tumors generated by subcutaneous implantation of MB49 cells were scanned once their diameter reached 10 mm, while orthotopic tumors were scanned when their volume exceeded 100 mm^3^. Tumor volume was calculated using the formula: Volume = length × width × height × (π / 3). All mice were anesthetized with isoflurane, with the dosage dynamically adjusted within the range of 0.5 to 1.8% to maintain a breathing rate between 30 and 40 breaths per minute throughout the experiment. All MR scans were conducted on a Bruker BioSpec 117/16 USR 11.7T animal system (Bruker, Ettlingen, Germany). A home‐built surface ^2^H/^1^H coil was used for both signal transmission and reception. T2‐weighted ^1^H anatomical axial images were first acquired to identify the tumor in all mice. The imaging parameters used were: repetition time (TR)/echo time (TE) = 3000/30 ms, matrix = 320 × 340, field of view (FOV) = 32 × 34 mm^2^, slice thickness = 2.5 mm, with 8 slices and 1 average.

3D ^2^H chemical shift imaging (CSI) was performed to evaluate deuterium‐labeled metabolites in subcutaneous and orthotopic bladder tumor models. The imaging parameters were as follows: TR/TE = 150/1.049 ms, matrix = 16 × 12 × 8, FOV = 32 × 24 × 20 mm^3^, bandwidth = 2000 Hz, flip angle (FA) = 60°, 256 spectral points, and 80 signal averages, resulting in an acquisition time of 20.5 min. After baseline acquisition, [2,3,4,6,6′‐^2^H_5_]‐D‐glucose (Dingbang Biotechnology, China) or [6,6′‐^2^H_2_]‐D‐glucose (Dingbang Biotechnology, China) was administered via the tail vein (3 g kg^−1^) or [2,3,4,6,6′‐^2^H_5_]‐D‐glucose was infused through a percutaneous catheter at a dose of 3 or 7.5 g kg^−1^ over 2 min using an infusion pump (Harvard Apparatus, USA). The imaging sequence was repeated five times—once pre‐infusion and four times post‐infusion. For the 3D‐CSI data, all spectral data were exported via TopSpin (4.2.0, Bruker, Germany) for post‐processing and analysis. Initially, zero‐padding, phase, and baseline corrections were applied to each spectrum. A Lorentzian model was then fitted, and the signal intensity of these metabolites was normalized to the signal intensity of ^2^H‐water at natural abundance before glucose infusion. The metabolite maps were subsequently interpolated to match the matrix size of the T2‐weighted images using Fourier transform‐based interpolation. All data processing was performed in MATLAB (R2023b, MathWorks, MA, USA).

### Diffusion Weighted and Dynamic Contrast‐Enhanced MRI

A separate cohort of percutaneous tumor‐bearing animals underwent diffusion‐weighted imaging (DWI) (n = 3) and dynamic contrast‐enhanced (DCE)‐MRI (n = 3) before and 24 h after the final chemotherapy treatment. All MRI experiments were performed using a 9.4 Tesla, 30‐cm‐diameter‐bore magnet (uMR 9.4T, United Imaging Life Science Instrument, P.R. China) equipped with a gradient insert of maximum strength 1000mT/m and a slew rate up to 10000T/m/s. An 86‐mm volume coil was used for transmission, and a three‐channel Rat Brain Surface Coil (RBSC3) was used for reception. Tumors were localized on axial fat‐suppressed T2‐weighted images acquired with a fast spin echo pulse sequence with the following parameters: TR/TE = 3000/ 39.72 ms, matrix = 336 × 100, FOV = 35 × 15 mm^2^, slice thickness = 0.6 mm. Diffusion‐weighted ^1^H images were acquired using an echo planar imaging sequence with the following parameters: TR/TE = 4800/15.5 ms, matrix = 140 × 100, FOV = 35 × 15 mm^2^, slice thickness = 0.6 mm, and 3 orthogonal diffusion directions with two b‐values (b = 200, 1000 s mm^−2^). Eight averages were acquired at b = 200 s mm^−2^, and 10 averages were acquired at b = 1000 s mm^−2^. The apparent diffusion coefficient (ADC) was measured prior to treatment and at 24 h post‐treatment in a 0.6‐mm‐thick slice covering the tumor. For DCE imaging, a 3D T1‐weighted gradient echo sequence was used with the following parameters: TR/TE = 5/2.08 ms, matrix = 176 × 100, FOV = 35 × 15 mm^2^, bandwidth (BW) = 300 Hz/pixel, slice thickness = 0.6 mm, and 16 slices. Baseline and dynamic axial T1 images were acquired following tail vein injection of ProHance at 200 mmol kg^−1^ (Gadoteridol, Bracco). Signals from the image series were converted, on a pixel‐by‐pixel basis, to a contrast‐agent concentration by assuming an R1 relaxivity of the contrast agent of 2.7 s^−1^(mmol/L)^−1^.^[^ ^39^ ^]^

### Histology and Immunohistochemistry

Tumors were fixed in 10% formalin for 24 h and then transferred to 70% ethanol. Samples were embedded in paraffin, sectioned at a thickness of 10 µm, and stained with hematoxylin and eosin (H&E). Immunohistochemical staining for Ki‐67 and cleaved caspase‐3 (CC3) was also carried out. The Ki‐67 antibody (Agilent; RRID: GB111141) was used at a dilution of 1:500, and the CC3 antibody (servicebio; RRID: GB11532) was used at a dilution of 1:600. Slides were scanned at 20× magnification with a resolution of 0.5 µm/pixel using an Aperio AT2 scanner (Leica Biosystems; RRID: SCR_02 1256). Images were analyzed using Qupath (version 0.5.1) to quantify the percentage of positively stained cells.

### Statistical Analysis

Statistical and graphical analyses were performed using Prism v9 (Graphpad, USA). Data were shown as mean ± standard deviation, unless stated otherwise. An analysis of variance (ANOVA) was performed for multiple comparisons among groups to determine significance. For single‐parameter comparisons, paired or unpaired Student *t* tests were performed, with errors represented as SD. All analyses were conducted using two‐tailed tests, and a *p*‐value below 0.05 was considered statistically significant.

## Conflict of interest

The authors declare that there are no competing interests.

## Supporting information



Supporting Information

## Data Availability

The data that support the findings of this study are available from the corresponding author upon reasonable request.
