# Supplementary material for: Early Detection of Chemotherapy‐Induced Glucose Metabolic Alterations in Bladder Carcinoma Using Deuterium (2H) Metabolic Imaging: in Vitro and in Vivo Assessments
Source: Adv Sci (Weinh). 2025 Nov 14;13(6):e14614. doi: 10.1002/advs.202514614 (PMC12866816; doi:10.1002/advs.202514614)
Supplement: Supplementary file 1 — Supporting Information [file ADVS-13-e14614-s001.docx]

**Supporting Information**

**Early Detection of Chemotherapy-Induced Glucose Metabolic Alterations in Bladder Carcinoma using Deuterium (²H) Metabolic Imaging: In Vitro and In Vivo Assessments**

*Lingmin Kong*, *Ganghan Yang*, *Bei Weng*, *Zhihua Wen*, *Joshua D. Kaggie*, *Qian Cai*, *Qian Wan*, *Cuien Zheng*, *Feng Du*, *Ye Li*, *Xin Liu*, *Ferdia A. Gallagher*, *Yan Guo*^*^, *Shi-Ting Feng*^*^, *Chao Zou*^*^, *Huanjun Wang* ^*^


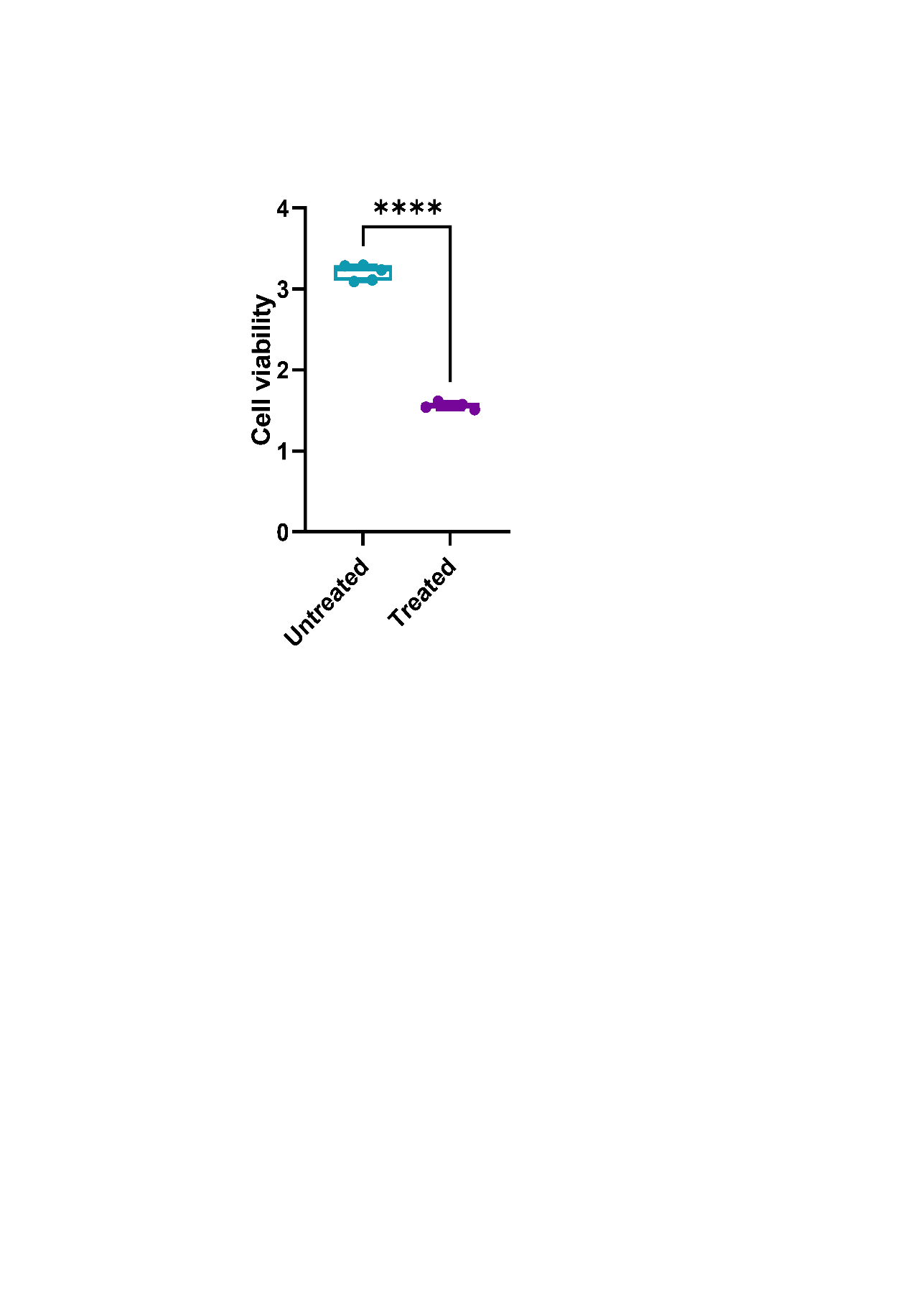


**Figure S1. Cell viability assessed using the CCK-8 assay.**  ****, *P* < 0.0001.

**
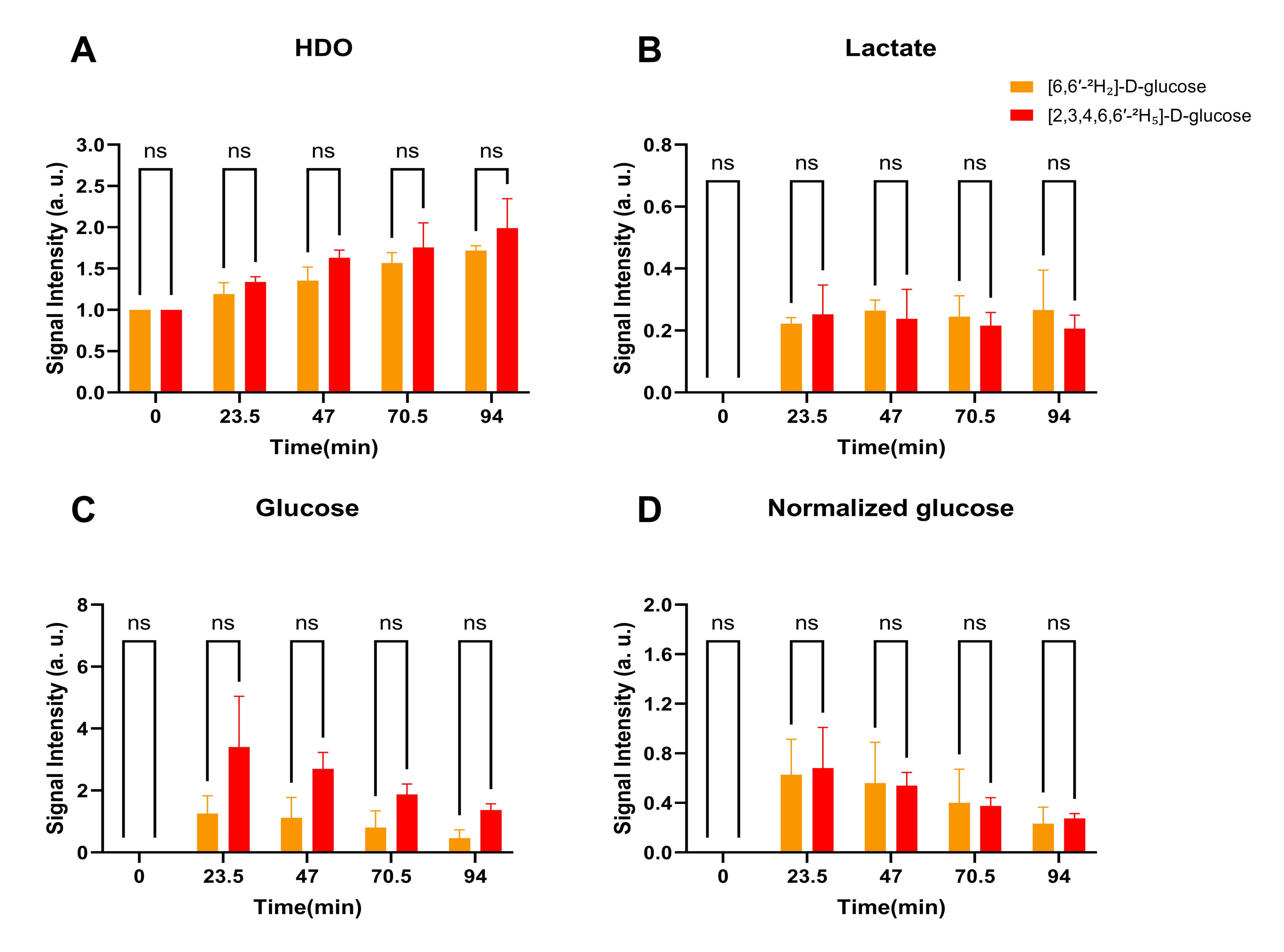
**

**Figure S2. Comparison of deuterium-labeled metabolite concentrations in MB49 subcutaneous tumor models using different tracers.** (A–D) Concentrations of ²H-labeled water (A), lactate (B), glucose (C) and normalized glucose (D) following administration of [6,6′-²H_2_]- (n = 3) or [2,3,4,6,6′-²H₅]-D-glucose (n = 4) via intravenous injection at a dose of 3 g/kg.


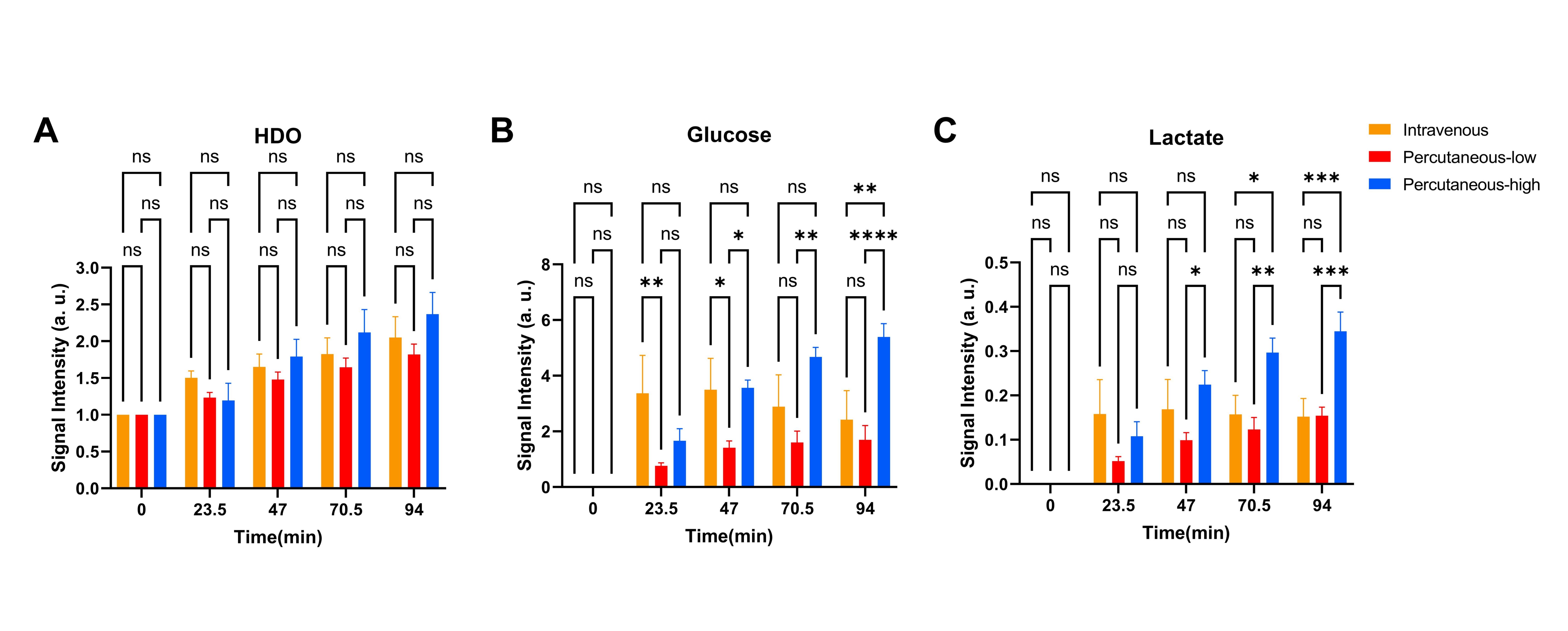


**Figure S3. Comparison of deuterium-labeled metabolite concentrations in MB49 subcutaneous tumor models using various administration routes and doses.** (**A–C**) Concentrations of ²H-labeled water (**A**), glucose (**B**), and lactate (**C**) following administration of [2,3,4,6,6′-²H₅]-D-glucose via intravenous injection at 3 g/kg (n = 5) or subcutaneous injection at 3 g/kg (n = 6) and 7.5 g/kg (n = 6).


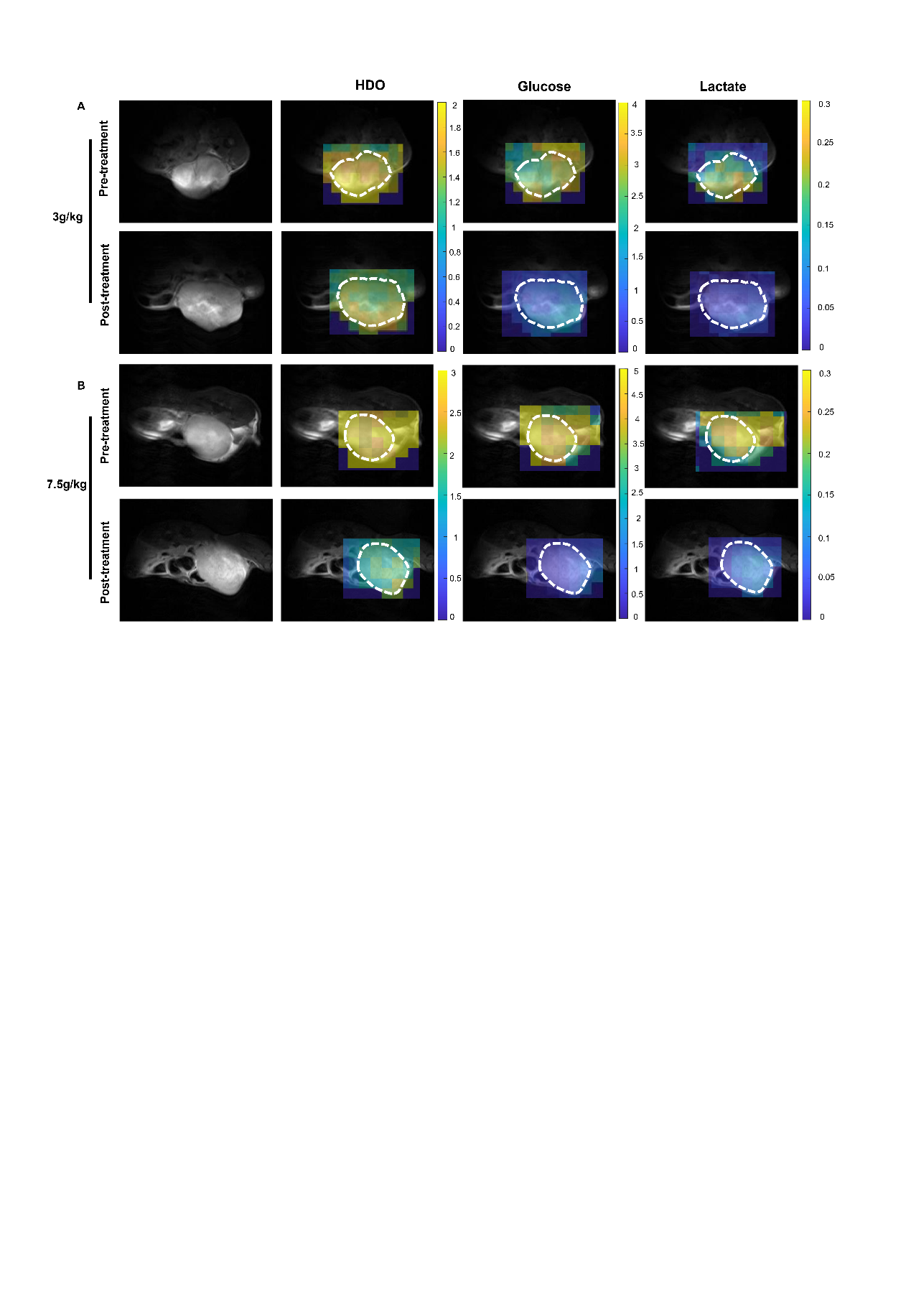


**Figure S4. Sensitivity of [2,3,4,6,6**′**-^2^H_5_]-D-Glucose metabolism to tumor metabolic activity**. (**A and B**) Images acquired from two animals before and after treatment, with the tumor region delineated by a white dashed line.


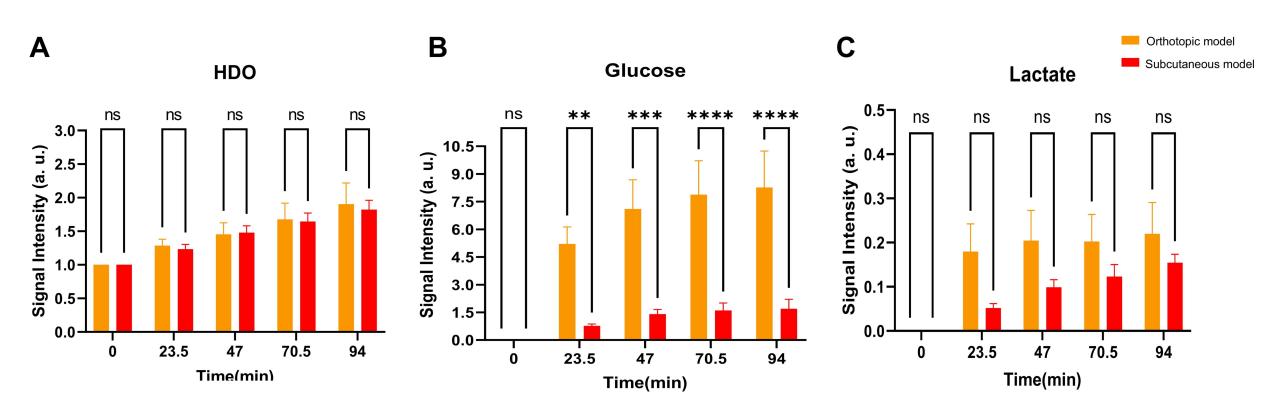


**Figure S5 Comparative analysis of ²H-labeled metabolites between MB49 subcutaneous and orthotopic tumor models.** (A-C) Comparison of ²H-labeled water (A), glucose (B) and lactate (C) between the two animal models at corresponding time points. ns, not significant; **, *P* < 0.01; ***, *P* < 0.001; and ****, *P* < 0.0001.
